# Development of Multi-Functional Chelators Based on Sarcophagine Cages

**DOI:** 10.3390/molecules19044246

**Published:** 2014-04-03

**Authors:** Shuanglong Liu, Zibo Li, Peter S. Conti

**Affiliations:** Molecular Imaging Center, Radiology Department, University of Southern California, CA 90089, USA

**Keywords:** positron emission tomography (PET), sarcophagine, ^64^Cu, bifunctional chelator (BFC)

## Abstract

A new class of multifunctionalized sarcophagine derivatives was synthesized for ^64^Cu chelation. The platform developed in this study could have broad applications in ^64^Cu-radiopharmaceuticals.

## 1. Introduction

**Scheme 1:**
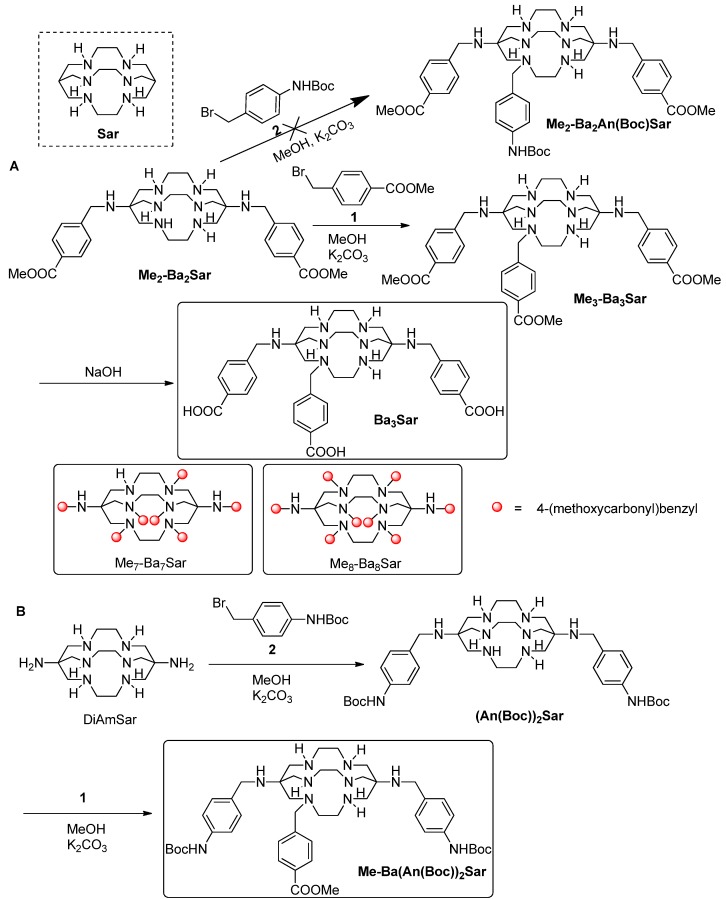
Synthesis of Ba_3_Sar, Ba_7_Sar, Ba_8_Sar and Me-Ba(An(Boc))_2_Sar.

Positron emission tomography (PET) is a powerful imaging modality to obtain quantitative molecular and biochemical information about physiological processes *in vivo*. With a half life of 12.7 h, copper-64 is well suited for the radiolabeling of proteins, antibodies, and peptides. Moreover, copper-64 decays by β^+^ (17.8%) and β^−^ emission (38.4%), making it an attractive radioisotope for both PET imaging (β^+^) and therapy (β^+^ and β^−^). Because direct conjugation of ^64^Cu onto bioligands is not practical, various bifunctional chelators (BFCs) have been developed for radiolabeling. Among all BFCs, macrocyclic chelators have demonstrated enhanced *in vivo* stability over acyclic chelators such as ethylenediaminetetraacetic acid (EDTA) and diethylenetriaminepentaacetic acid (DTPA). Up to now, a number of macrocyclic BFCs have been developed and applied in PET probe designs, including 1,4,7,10-tetraazacyclododecane-*N*,*N*',*N*'',*N*'''-tetraacetic acid (DOTA) [1,2], 1,4,7-triaza-cyclononane-1,4,7-triacetic acid (NOTA) derivatives [3,4], cross-bridged 1,4,8,11-tetraazacyclo-tetradecane-1,4,8,11-tetraacetic acid (CB-TETA) [2,5], and 1,4,8,11-tetraazabicyclo[6.6.2]hexadecane (CB-TE2A) [6,7,8]. In particular, various studies have demonstrated that the hexaazamacrobicyclic sarcophagines (denoted as “Sar”, Scheme 1) could form extraordinarily stable Cu complexes under mild conditions with good *in vivo* stability [9,10,11,12,13,14,15,16].

Recently, we successfully improved the functionalization approach of the Sar cage through a direct alkylation (SN_2_) reaction and synthesized BaBaSar that has two pendant carboxylate groups at either end of the Sar cage. This novel multivalency bifunctional chelator could be further conjugated to multiple targeting ligands (such as RGD peptides) via biologically stable amide bonds. Unlike monovalent ligands, the polyvalent ligands could lead to increased target binding affinity and tumor uptake [17,18,19,20,21,22,23]. For example, dimeric, tetrameric and octameric RGD peptides (integrin α_v_β_3_ targeting) have been developed to increase tumor uptake based on polyvalency principle [18,19,24]. Clearly, a Sar cage with multiple functional groups for further modification could serve as an important platform for the construction of multivalent probes. Herein, we report a streamlined synthetic approach to the multi-functionalized Sar cage suitable for the design and syntheses of polyvalent probes. We also explore the *in vitro* and *in vivo* stability of ^64^Cu labeled Ba_3_Sar.

## 2. Results and Discussion

Our initial syntheses are shown in Scheme 1. The precursor Me_2_-Ba_2_Sar was synthesized as reported [23]. Further alkylation of Me_2_-Ba_2_Sar was realized using methyl 4-bromomethylbenzoate (**1**) in the presence of Na_2_CO_3_ as base. MeOH was used as the solvent due to its good solubilization of Sar cages. Due to the low selectivity of the multiple amino groups on Me_2_-Ba_2_Sar, the reaction mixture was complicated, as indicated by HPLC. However, we could control the ratio of Me_2_-Ba_2_Sar and **1** to achieve reasonable yields for the desired products. For example, in the synthesis of Me_3_-Ba_3_Sar, we used a 1.2:1 ratio of **1** to Me_2_-Ba_2_Sar, and Me_3_-Ba_3_Sar was obtained in the yield of 32%. When 10 equivalent of **1** was used for the synthesis, Me_7_-Ba_7_Sar and Me_8_-Ba_8_Sar could be obtained in the yields of 15% and 12%, respectively. The methyl protecting groups were readily removed in 0.2 N NaOH to afford Ba_3_Sar, Ba_7_Sar, and Ba_8_Sar in almost quantitative yields. The free benzoic acids are useful for the conjugation with terminal or lysine side chain amino groups of peptides or proteins. In addition to the homo-functionalized Sar cages, it would be interesting to introduce different functional groups to the Sar cages. However, our initial test on modifying Me_2_-Ba_2_Sar with *tert*-butyl(4-(bromomethyl)phenyl) carbamate (**2**) failed to provide us the heterofunctionalized Me_2_-Ba_2_An(Boc)Sar (Scheme 1), which may be attributed to the reactivity difference between methyl 4-bromomethylbenzoate and *tert*-butyl(4-(bromomethyl)phenyl)carbamate. In order to obtain a heterofunctionalized sarcophagine, we synthesized the protected (An(Boc))_2_Sar as our previous report (Scheme 1). The purified (An(Boc))_2_Sar was then further alkylated with **1** to give Me-Ba(An(Boc))_2_Sar with Boc and methyl protective groups on it. The overall yield for Me-Ba(An(Boc))_2_Sar from DiAmSar was 22%.

**Figure 1:**
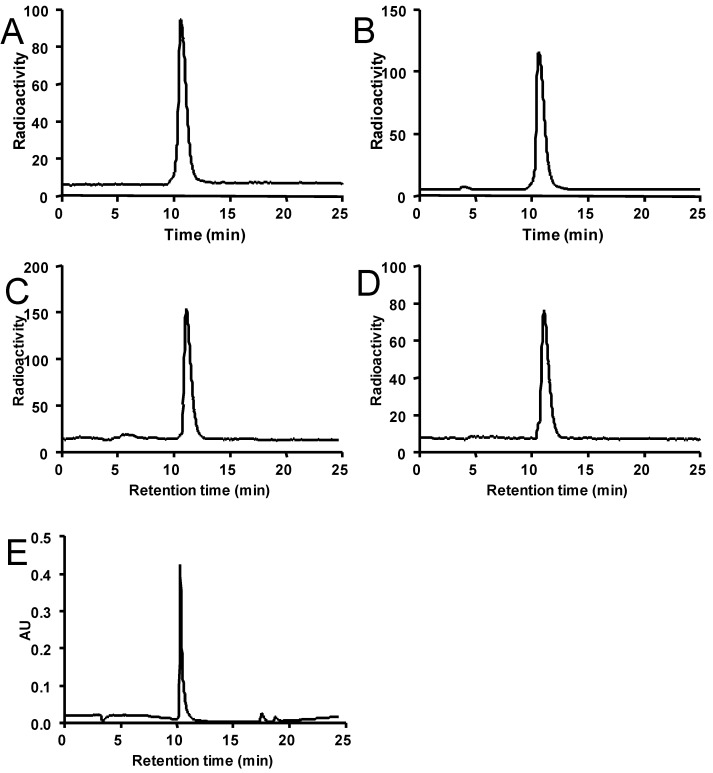
*In vitro* stability. (**A**) The radiotrace standard of ^64^Cu-Ba_3_Sar. (**B**) The radiotrace of ^64^Cu-Ba_3_Sar after 20 h incubation in 1 × PBS. (**C**) The radiotrace of ^64^Cu-Ba_3_Sar after 3 h incubation in mouse serum. (**D**) The radiotrace of ^64^Cu-Ba_3_Sar after 24 h incubation in mouse serum. (**E**) The UV trace of standard ^64^Cu-Ba_3_Sar.

As a proof of principle experiment, we chose Ba_3_Sar as an example to test the radiolabeling efficacy of the synthesized chelators. As shown in Figure 1, Ba_3_Sar can be efficiently labeled with ^64^Cu at pH 5.5 in sodium acetate buffer after 20 min incubation at 40 °C. Even without purification, radio trace HPLC showed greater than 95% purity of ^64^Cu-Ba_3_Sar. Furthermore, to broaden the application of Ba_3_Sar, we tested the ^64^Cu labeling in basic conditions, potentially useful for bioligands sensitive to acids. In phosphate buffer (pH 7.4) and borate buffer (pH 8.5), the radiolabeling of Ba_3_Sar could give 71% and 81% yield after 30 min incubation at 40 °C, respectively. To estimate the highest achievable limit of the specific activity of the product, we gradually decreased the amount of Ba_3_Sar added to the reaction. When 2 µg Ba_3_Sar was loaded into 2 mCi (74 MBq) ^64^Cu solution, the labeling yield was still as high as 75%. The specific activity of the radiolabeled conjugate was 500 mCi/µmol.

The *in vitro* stability of ^64^Cu-Ba_3_Sar was evaluated after incubation in 1 × PBS and 10% mouse serum by radio HPLC (Figure 1). No significant amount of free ^64^Cu was detected by radio HPLC up to 20 h post incubation. We also like to point out that less than 5% of ^64^Cu was trapped on NanoSep 10 K filter, suggesting minimum activity was trapped in serum proteins. These data are consistent with the previously published stability results [9,10,11,12,13]. The cross-bridged and cage-like configuration of the Sar structure could lead to the high stability of the complex. 

**Figure 2:**
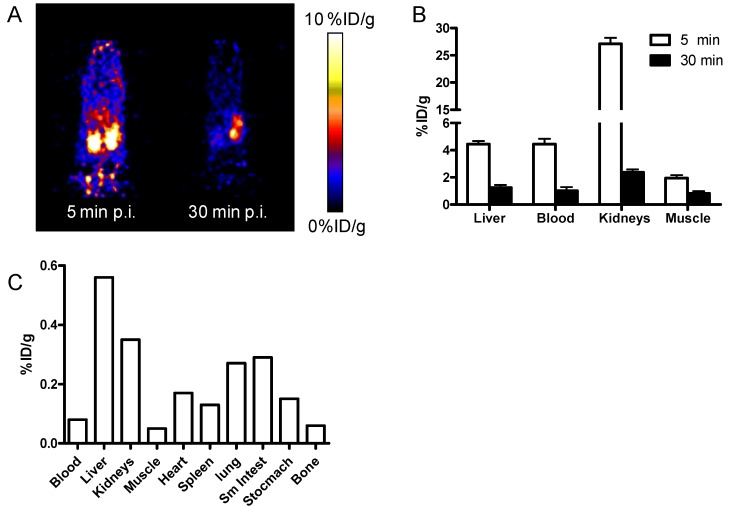
(**A**) Decay-corrected whole-body coronal microPET images of athymic female nude mice from a static scan at 5 min, and 30 min after injection of ^64^Cu-Ba_3_Sar. (**B**) microPET quantification of major organs at 5 min, and 30 min after injection of ^64^Cu-Ba_3_Sar. The data are expressed as average ± standard deviation, *n* = 3. (**C**) Biodistribution studies of ^64^Cu-Ba_3_Sar in normal female nude mouse at 24 h after injection.

The *in vivo* distribution and stability of ^64^Cu-Ba_3_Sar were evaluated by static microPET scans at 5 min and 30 min after injection of ^64^Cu-Ba_3_Sar via tail vain into 6–7 weeks old nude mice (Figure 2). microPET images show that the activity is fast cleared from kidneys. At 5 min post injection, the liver uptake is 4.45 ± 0.40%ID/g, which has no significant difference compared with blood uptake (4.38 ± 0.67%ID/g). At 30 min p.i., the liver uptake (1.26 ± 0.32%ID/g) is only slightly higher than the blood (1.02 ± 0.47%ID/g) and muscle (0.83 ± 0.28%ID/g). The low liver uptake and fast clearance from body indirectly suggested the high *in vivo* stability of ^64^Cu-Ba_3_Sar since the released free ^64^Cu would be easily accumulated in liver [25]. To further investigate the localization of ^64^Cu-Ba_3_Sar in normal athymic nude mice, we also performed biodistribution study at 24 h after injection. As can be seen in Figure 2C, the highest uptake was found in liver (0.56%ID/g). The low uptake in normal organs also suggests the high *in vivo* stability of ^64^Cu-Ba_3_Sar. Overall, the circulation of ^64^Cu-Ba_3_Sar is similar to our previous investigated sarcophagine derivatives.

## 3. Experimental

### 3.1. General

All chemicals obtained commercially were of analytic grade and used without further purification. The syringe filter and polyethersulfone membranes (pore size, 0.22 μm; diameter, 13 mm) were obtained from Nalge Nunc International (Rochester, NY, USA). The semi-preparative reversed-phase HPLC using a Vydac protein and peptide column (218TP510; 5 µm, 250 × 10 mm) was performed on a Dionex 680 chromatography system with a UVD 170U absorbance detector (Sunnyvale, CA, USA) and model 105S single-channel radiation detector (Carroll & Ramsey Associates, Berkeley, CA, USA). With a flow rate of 4 mL/min, the mobile phase stayed at 95% solvent A [0.1% trifluoroacetic acid (TFA) in water] and 5% B [0.1% TFA in acetonitrile (MeCN)] at 0–2 min and was changed from 95% solvent A and 5% B at 2 min to 35% solvent A and 65% solvent B at 32 min. Analytical HPLC had the same gradient with flow rate of 1 mL/min using a Vydac protein and peptide column (218TP510; 5 µm, 250 × 4.6 mm). The UV absorbance was monitored at 218 nm and the identification of the peptides was confirmed based on the UV spectrum using a PDA detector. Copper-64 was purchased from University of Wisconsin. It was dissolved in 0.1 N HCl solution and the specific activity > 1 Ci/µmol. For all the solvents used in the labeling study, Chelex 100 resin was used to remove heavy metal. MicroPET scans were performed on a microPET R4 rodent model scanner (Siemens Medical Solutions USA, Inc., Knoxville, TN, USA). The scanner has a computer-controlled bed and 10.8-cm transaxial and 8-cm axial fields of view (FOVs). It has no septa and operates exclusively in the 3-dimensional (3D) list mode. Animals were placed near the center of the FOV of the scanner. ^1^H and ^13^C NMR spectra were obtained on a 300 MHz superconducting NMR spectrometer (Bruker, Billerica, MA, USA). The mass spectra were recorded by Electrospray Ionization Mass spectrum (Shimadzu, Pleasanton, CA, USA).

### 3.2. Preparation of Ba_3_Sar, Ba_7_Sar, and Ba_8_Sar

Me_2_-Ba_2_Sar was synthesized as reported in the literature [9,10,11]. To a solution of Me_2_-Ba_2_Sar (38.9 mg, 63.7 µmol) in MeOH (5 mL), was added 1.2 equiv. of methyl 4-(bromomethyl)benzoate (1, 17.0 mg, 76.4 µmol) in tetrahydrofuran (THF, 2.5 mL) and sodium carbonate (Na_2_CO_3_, 31.8 mg, 300 µmol). The reaction was refluxed under stirring for 5 h. After cooling down to room temperature, 5% acetic acid (5 mL) was added the crude mixture. Semipreparative HPLC afforded the Me_3_-Ba_3_Sar as off-white solid (32%, 15.4 mg, 20.4 µmol). The retention time of Me_3_-Ba_3_Sar on analytical HPLC is 20.6 min. The molecular weight was determined by the Electrospray Ionization Mass spectrum (ESI-MS) to be 759.4 for [M+H]^+^ (chemical formula: C_41_H_59_N_8_O_6_, calculated molecular weight: 759.5). To remove the methyl protection groups from Me_3_-Ba_3_Sar, Me_3_-Ba_3_Sar (5 mg, 6.6 µmol) was dissolved in 0.2 N NaOH (1 mL) and the reaction mixture was kept at 60 °C for 1 h. After cooling down to room temperature, 5% acetic acid (5 mL) was added the crude mixture. Semipreparative HPLC afforded Ba_3_Sar as white powder (98%, 4.6 mg, 6.4 µmol). The retention time of Ba_3_Sar on analytical HPLC is 10.4 min. The molecular weight of Ba_3_Sar was determined by the Electrospray Ionization Mass spectrum (ESI-MS) to be 717.3 for [M+H]^+^ (chemical formula: C_38_H_53_N_8_O_6_, calculated molecular weight: 717.4). ^1^H-NMR (300 MHz, DMSO-*d*_6_): δ = 13.1 (s, 3H), 7.94 (d, *J* = 8.0 Hz, 8H), 7.51 (d, *J* = 7.3 Hz, 4H), 3.92–3.32 (m, 18H), 3.21–2.07 (m, 18H). ^13^C-NMR (75 MHz, DMSO-*d*_6_): δ = 167.2, 167.1, 158.6, 158.2, 158.0, 144.0, 130.1, 130.0, 129.4, 118.2, 115.2, 59.1, 57.2, 55.6, 45.1, 44.3. The molecular weight of Ba_7_Sar was determined by the Electrospray Ionization Mass spectrum (ESI-MS) to be 1253.7 for [M+H]^+^ (chemical formula: C_70_H_77_N_8_O_14_, calculated molecular weight: 1253.6). The molecular weight of Ba_8_Sar was determined by the Electrospray Ionization Mass spectrum (ESI-MS) to be 1387.8 for [M+H]^+^ (chemical formula: C_78_H_83_N_8_O_16_, calculated molecular weight: 1387.6).

### 3.3. Prepareation of Ba(An(Boc))_2_Sar

To the solution of DiAmSar (20 mg, 63.7 µmol) in MeOH (20 mL) was added 4-(bromomethyl)benzoate (**1**, 43.5 mg, 191.1 µmol) solution in THF (10 mL) and Na_2_CO_3_ (21.2 mg, 200 µmol). The reaction was refluxed under stirring for 10 h. After cooling down to room temperature, 5% acetic acid (25 mL) was added the crude mixture. Semipreparative HPLC afforded the (An(Boc))_2_Sar (44%, 20.3 mg, 28.0 µmol) as a white solid. The molecular weight for An_2_Sar was determined by the Electrospray Ionization Mass spectrum (ESI-MS) to be 725.3 for [M+H]^+^ (chemical formula: C_38_H_65_N_10_O_4_, calculated molecular weight: 725.5).

To further alkylate (An(Boc))_2_Sar to Me-Ba(An(Boc))_2_Sar, (An(Boc))_2_Sar (5 mg, 6.9 µmol) dissolved in MeOH (0.5 mL) was added to methyl 4-(bromomethyl)benzoate (**1**, 3.1 mg, 13.8 µmol) in THF (1 mL) and Na_2_CO_3_ (1.6 mg, 15 µmol). The reaction mixture was refluxed for 5 h. After cooling down to room temperature, 5% acetic acid (5 mL) was added the crude mixture. Semipreparative HPLC afforded the Me-Ba(An(Boc))_2_Sar as white powder (50%, 3.0 mg, 3.4 µmol). The retention time of Me-Ba(An(Boc))_2_Sar on analytical HPLC is 12.7 min. The molecular weight was determined by the Electrospray Ionization Mass spectrum (ESI-MS) to be 873.7 for [M+H]^+^ (chemical formula: C_47_H_73_N_10_O_6_, calculated molecular weight: 873.6).

### 3.4. Radiochemistry

*Acidic conditions:*^64^CuCl_2_ (20 µL, 74 MBq in 0.1 N HCl) was diluted in 0.1 N sodium acetate (80 µL, pH 5.5) and added to Ba_3_Sar (20 µg). The reaction mixture was kept at 40 °C for 20 min. ^64^Cu-labeled product was subsequently purified by analytical HPLC and the radioactive peak containing the desired product was collected. After removal of the solvent by rotary evaporation, the ^64^Cu-Ba_3_Sar tracer was reconstituted in PBS (1 mL) and passed through a 0.22 µm syringe filter for *in vivo* animal experiments. The decay-corrected radiochemical yield (RCY) was 95%.

*Basic conditions:* ^64^CuCl_2_ (20 µL, 74 MBq in 0.1 N HCl) was diluted in 0.1 N phosphate (80 µL, pH 7.4) or 0.1 N borate buffer (80 µL, pH 8.5) and added to Ba_3_Sar (20 µg). The reaction mixture was kept at 40 °C for 30 min. ^64^Cu-labeled product was subsequently purified by analytical HPLC and the radioactive peak containing the desired product was collected. The decay-corrected radiochemical yields (RCY) were 71% (pH 7.4) and 81% (pH 8.5).

### 3.5. In Vitro Stability

20 MBq ^64^Cu-Ba_3_Sar was incubated in 1× PBS (1 mL, pH 7.4) at 40 °C for 24 h. Then, the stability was measured by analytical radio HPLC with the above mentioned program.

### 3.6. Serum Stability of ^64^Cu-Ba_3_Sar

The *in vitro* stability of ^64^Cu-Ba_3_Sar was evaluated by incubation of 7.4 MBq (200 μCi) of ^64^Cu-Ba_3_Sar with mouse serum (10%, 1 mL) at 37 °C. At 3 h and 24 h, the solution was filtered through a NanoSep 10 K centrifuge (Pall Corp., Port Washington, NY, USA) to isolate the low-molecular-weight metabolites. The NanoSep 10 K filter was washed with PBS (200 µL) two more times. The filtrates were combined and analyzed by reverse-phase HPLC using conditions identical to those used for the standard ^64^Cu-Ba_3_Sar analysis.

### 3.7. MicroPET Imaging and Biodistribution

Animal procedures were performed according to a protocol approved by the University of Southern California Institutional Animal Care and Use Committee. For static microPET scans, the mice were injected with approximately 3.7 MBq (100 μCi) of ^64^Cu-Ba_3_Sar *via* the tail vein (*n* = 3 for each group). At 5 min and 30 min post injection (p.i.), the mice were anesthetized with isoflurane (5% for induction and 2% for maintenance in 100% O_2_) using a knock-down box. With the help of a laser beam attached to the scanner, the mice were placed in the prone position and near the center of the field of view of the scanner. The 3-min static scans were then obtained. Images were reconstructed by use of a 2-dimensional ordered-subsets expectation maximization (OSEM) algorithm. No background correction was performed. Regions of interest (ROIs; 5 pixels for coronal and transaxial slices) were drawn over the organs of interest on decay-corrected whole-body coronal images. The maximum counts per pixel per minute were obtained from the ROI and converted to counts per milliliter per minute by using a calibration constant. With the assumption of a tissue density of 1 g/mL, the ROIs were converted to counts per gram per min. Image ROI-derived %ID/g values were determined by dividing counts per gram per minute by injected dose. No attenuation correction was performed. The biodistribution study at 24 h post injection of ^64^Cu-Ba_3_Sar was performed in a normal female nude mouse as reported [26]. 

## 4. Conclusions

Novel homo-/hetero-functionalized sarcophagine chelators for ^64^Cu radiopharmaceuticals have been developed. In our initial evaluation, good *in vitro* and *in vivo* stability was observed for ^64^Cu-Ba_3_Sar. These multifunctional chelators could serve as a versatile building platform for multivalent/multimodaltity imaging probe construction, which would have a broad application in both imaging and therapy related research involving copper and other radiometals. 
